# Supplementary material for: Non-linear transcriptional responses to gradual modulation of transcription factor dosage
Source: bioRxiv. 2024 Mar 3:2024.03.01.582837. Preprint. [Version 1] doi: 10.1101/2024.03.01.582837 (PMC10925300; doi:10.1101/2024.03.01.582837)
Supplement: Supplementary file 1 [file NIHPP2024.03.01.582837v1-supplement-1.pdf]

bioRxiv preprint doi: https://doi.org/10.1101/2024.03.01.582837; this version posted March 4, 2024. The copyright holder for this preprint (which was not certified by peer review) is the author/funder, who has granted bioRxiv a license to display the preprint in perpetuity. It is made available under aCC-BY 4.0 International license.

Supplementary Figures

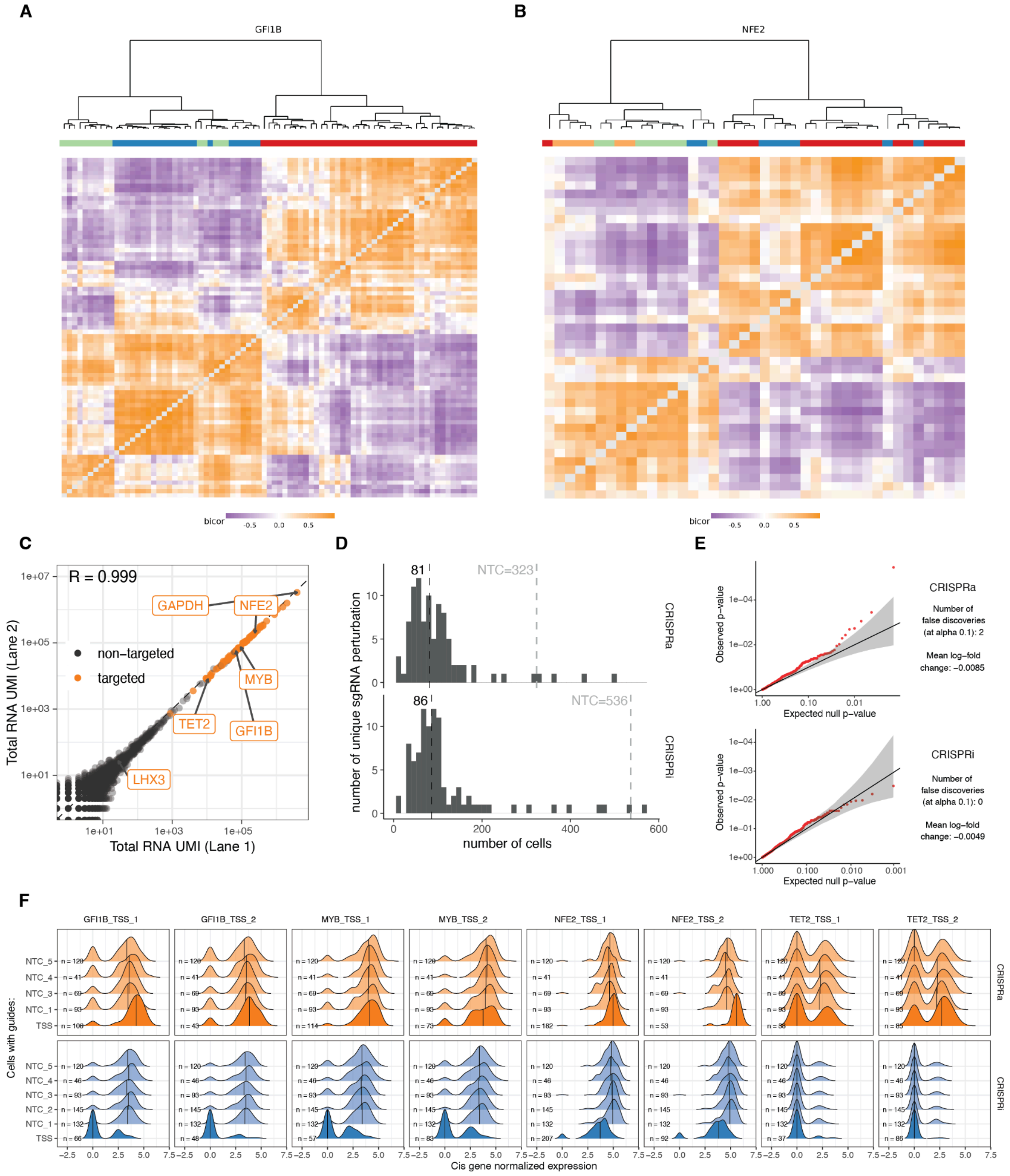

Figure S1: Experimental design and data processing from UMIs to expression fold-change, related to Figure 1 and STAR methods.

A. Co-expression matrix of the 76 selected GFI1B trans genes based on K562 data from [1]. Three clusters from the selected targeted panel show similar co-expression architecture than the original clusters identified using the entire GFI1B trans-network (original clusters A in blue, B in green and C in red).

bioRxiv preprint doi: https://doi.org/10.1101/2024.03.01.582837; this version posted March 4, 2024. The copyright holder for this preprint (which was not certified by peer review) is the author/funder, who has granted bioRxiv a license to display the preprint in perpetuity. It is made available under aCC-BY 4.0 International license.

B. Same as (A) for the 39 NFE2 trans genes (original clusters A in green, B in orange, C in blue and D in red).
C. Correlation between total UMI counts per gene between 10X chip lanes. Targeted panel genes are shown in orange and highlighted names correspond to dosage genes (NFE2, MYB, GFI1B and TET2) and low/high expression controls (LHX3 and GAPDH).
D. The number of singlet cells carrying each sgRNA in the two different CRISPR cell lines. NTC = non-targeting controls.
E. Q-Q plots from Sceptre calibration test.
F. Distribution of normalised UMI expression of the cis gene labelled on top for cells with sgRNAs targeting their TSS or harbouring NTC sgRNAs.

bioRxiv preprint doi: https://doi.org/10.1101/2024.03.01.582837; this version posted March 4, 2024. The copyright holder for this preprint (which was not certified by peer review) is the author/funder, who has granted bioRxiv a license to display the preprint in perpetuity. It is made available under aCC-BY 4.0 International license.

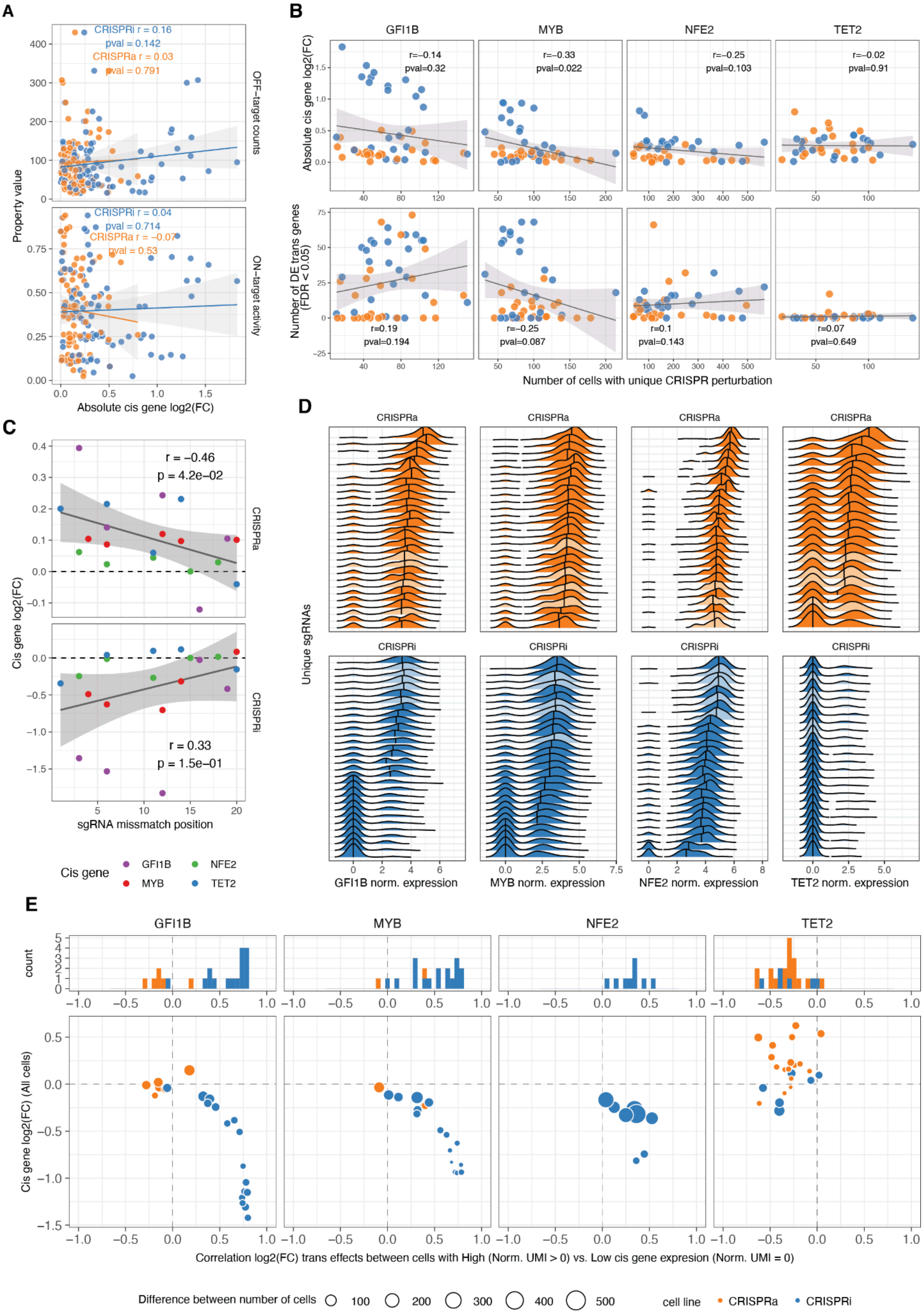

Figure S2: Biochemical and activity properties of different types of sgRNAs

bioRxiv preprint doi: https://doi.org/10.1101/2024.03.01.582837; this version posted March 4, 2024. The copyright holder for this preprint (which was not certified by peer review) is the author/funder, who has granted bioRxiv a license to display the preprint in perpetuity. It is made available under aCC-BY 4.0 International license.

A. Relationship between off-target and on-target activity of sgRNAs and the change in expression of their target cis gene.
B. Relationship between the number of cells that covered each sgRNA perturbation with the absolute fold change of the cis gene (top) or the number of differentially expressed trans genes due to the cis gene perturbation (bottom).
C. Relationship between the location of the mismatch mutation of attenuated sgRNAs (position 1 being farthest away from PAM motif location) and their effect on the cis gene expression.
D. Distribution of the normalised cis gene UMIs in single cells, grouped by their unique sgRNAs, ranked top to bottom by mean normalised expression. Transparent distributions correspond to non-targeting controls.
E. Distribution of the correlation in trans gene expression fold-changes when splitting the same sgRNA cells into 0 UMI or >0 UMI for the cis gene (top panel). Comparison of the strength of these correlations with the effect of that sgRNA on the cis gene (bottom panel). Size of dots indicate the difference in the size of the 0 UMI or >0 UMI cell groups.

bioRxiv preprint doi: https://doi.org/10.1101/2024.03.01.582837; this version posted March 4, 2024. The copyright holder for this preprint (which was not certified by peer review) is the author/funder, who has granted bioRxiv a license to display the preprint in perpetuity. It is made available under aCC-BY 4.0 International license.

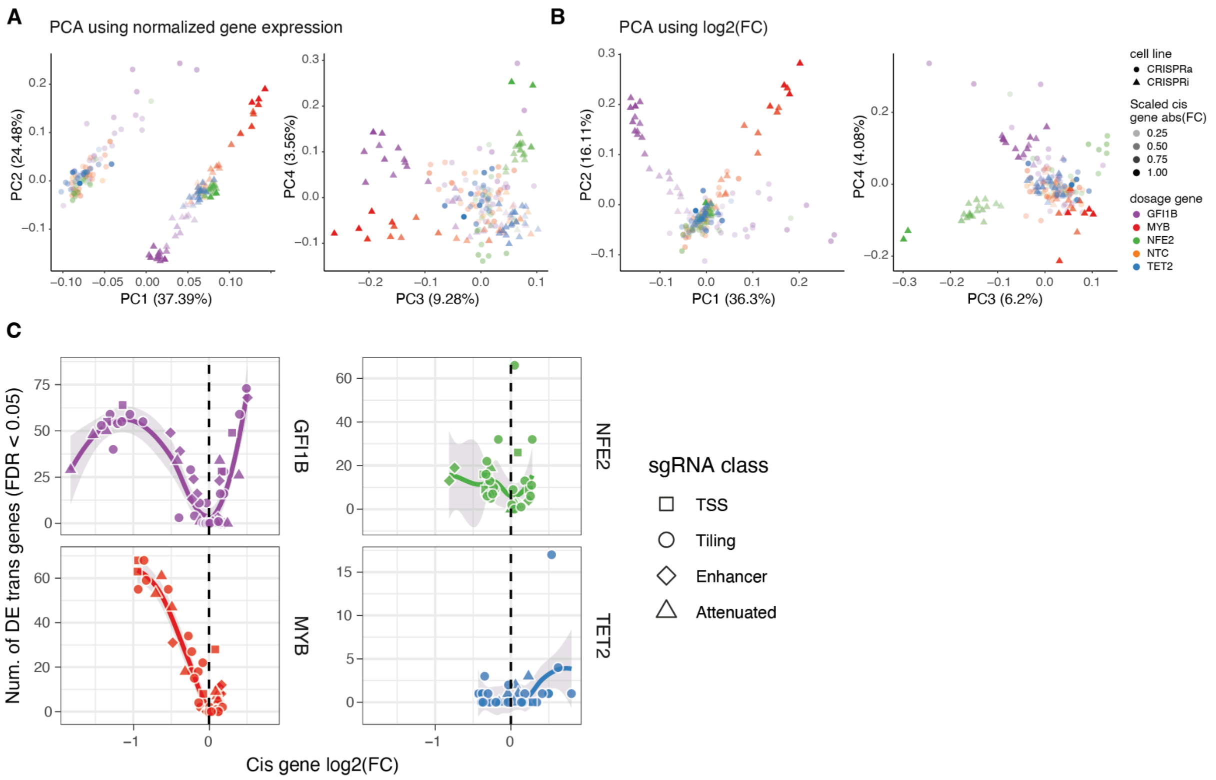

Figure S3: Global view of trans effects when modulating TF dosage

A. PCA of mean UMI normalised expression (not relative to each cell line of origin) for all genes across unique sgRNA perturbations.
B. Same as A but using relative expression fold-change when normalising by the CRISPR cell line of origin.
C. Number of differentially expressed trans genes relative to the cis gene dosage perturbation.

bioRxiv preprint doi: https://doi.org/10.1101/2024.03.01.582837; this version posted March 4, 2024. The copyright holder for this preprint (which was not certified by peer review) is the author/funder, who has granted bioRxiv a license to display the preprint in perpetuity. It is made available under aCC-BY 4.0 International license.

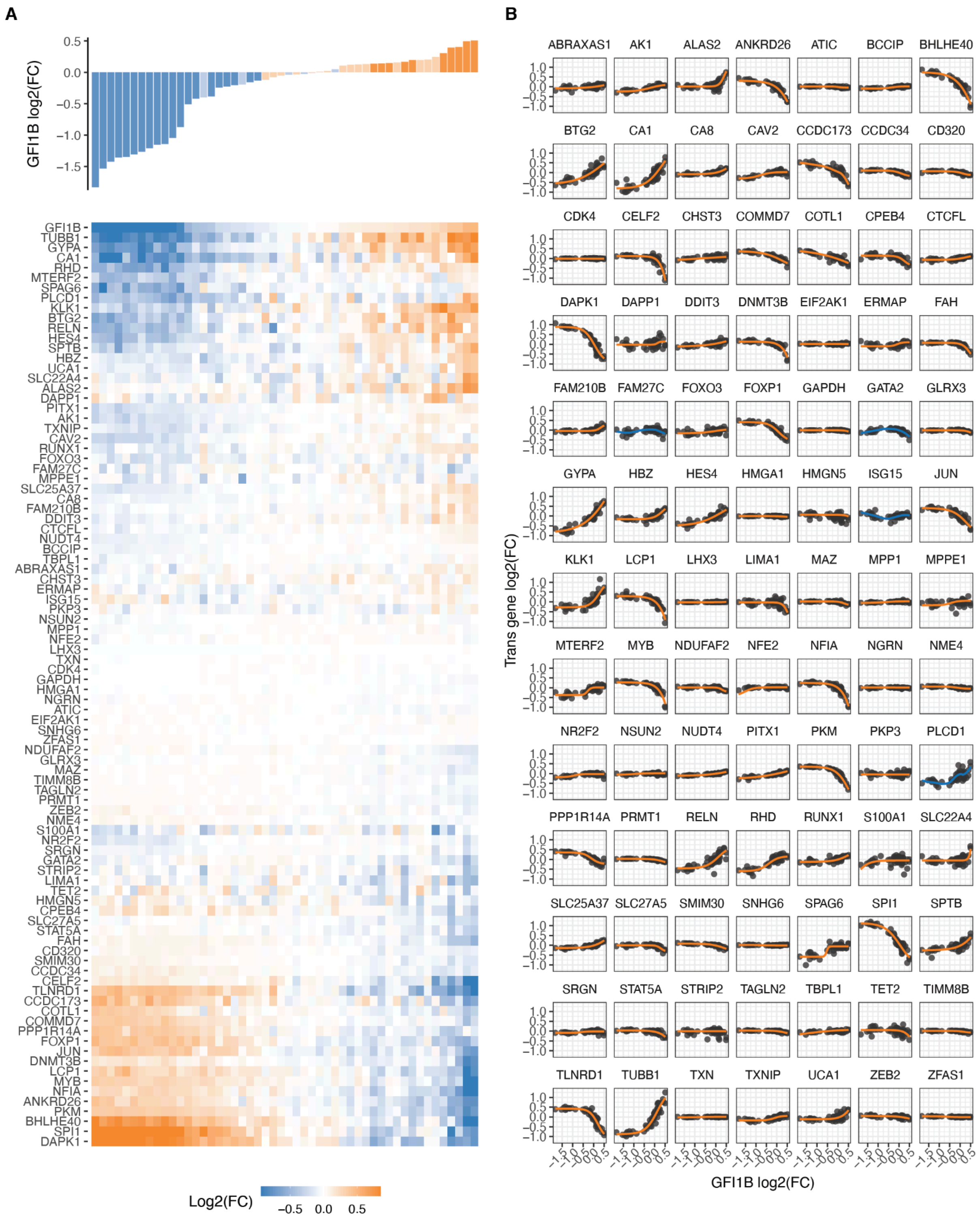

Figure S4: Transgenes responses to GFI1B dosage modulation

A. Changes in relative expression of all trans genes (heatmap) in response to GFI1B expression (top barplot) upon each distinct targeted sgRNA perturbation. The rows of the heatmap (trans genes) are hierarchically clustered based on their expression fold change linked to alterations in GFI1B dosage.

bioRxiv preprint doi: https://doi.org/10.1101/2024.03.01.582837; this version posted March 4, 2024. The copyright holder for this preprint (which was not certified by peer review) is the author/funder, who has granted bioRxiv a license to display the preprint in perpetuity. It is made available under aCC-BY 4.0 International license.

B. Dosage response curves are plotted for each trans gene against changes in GFI1B expression. The orange line represents the sigmoid model fit, and the blue line represents a loess curve.

bioRxiv preprint doi: https://doi.org/10.1101/2024.03.01.582837; this version posted March 4, 2024. The copyright holder for this preprint (which was not certified by peer review) is the author/funder, who has granted bioRxiv a license to display the preprint in perpetuity. It is made available under aCC-BY 4.0 International license.

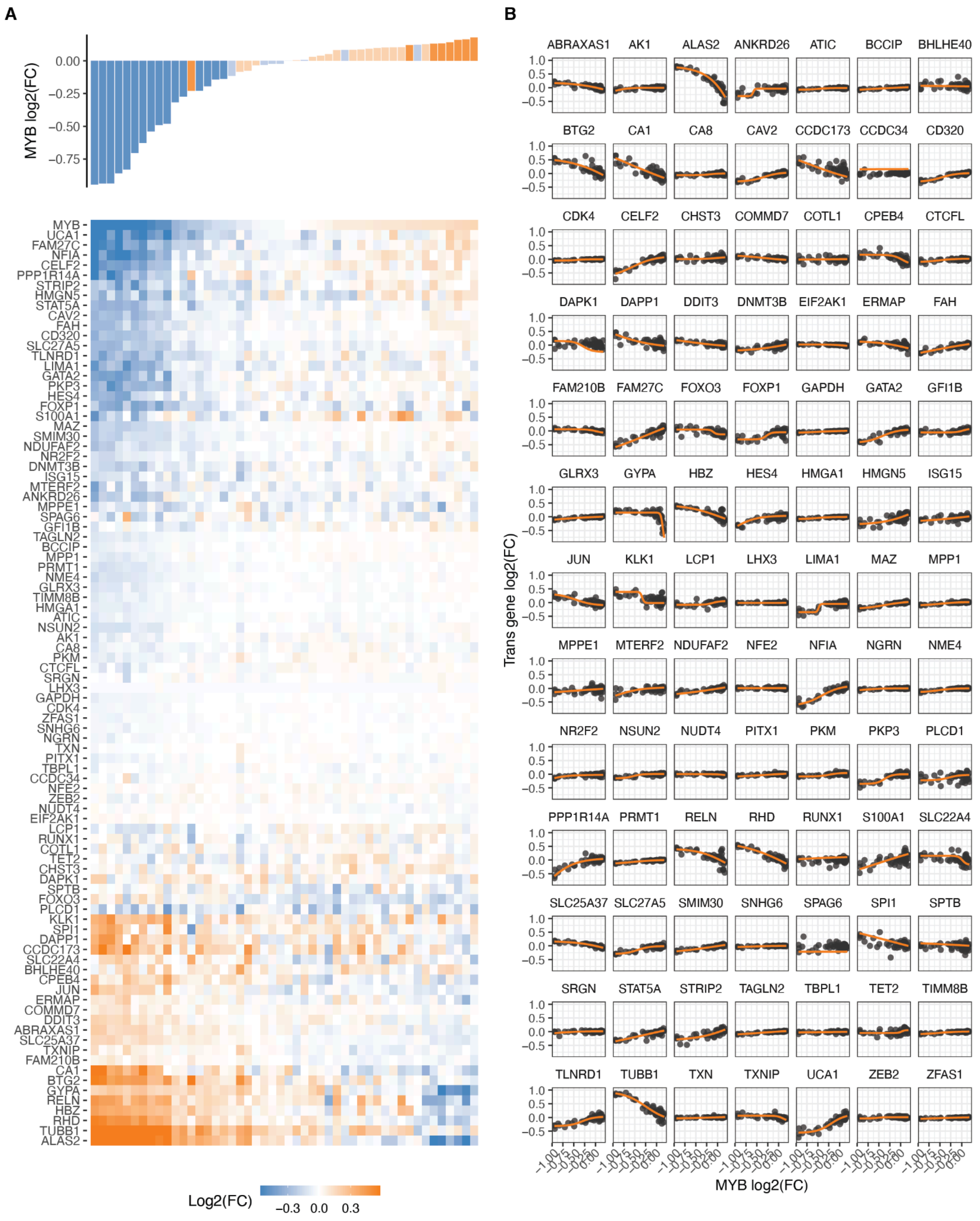

Figure S5: Transgenes responses to MYB dosage modulation

A. Changes in relative expression of all trans genes (bottom heatmap) in response to MYB expression (top barplot) upon each distinct targeted GFI1B sgRNA perturbation. The rows of the heatmap (trans genes) are hierarchically clustered based on their expression fold change linked to alterations in MYB dosage.
B. Dosage response curves are plotted for each trans gene against changes in MYB expression. The orange line represents the sigmoid model fit.

bioRxiv preprint doi: https://doi.org/10.1101/2024.03.01.582837; this version posted March 4, 2024. The copyright holder for this preprint (which was not certified by peer review) is the author/funder, who has granted bioRxiv a license to display the preprint in perpetuity. It is made available under aCC-BY 4.0 International license.

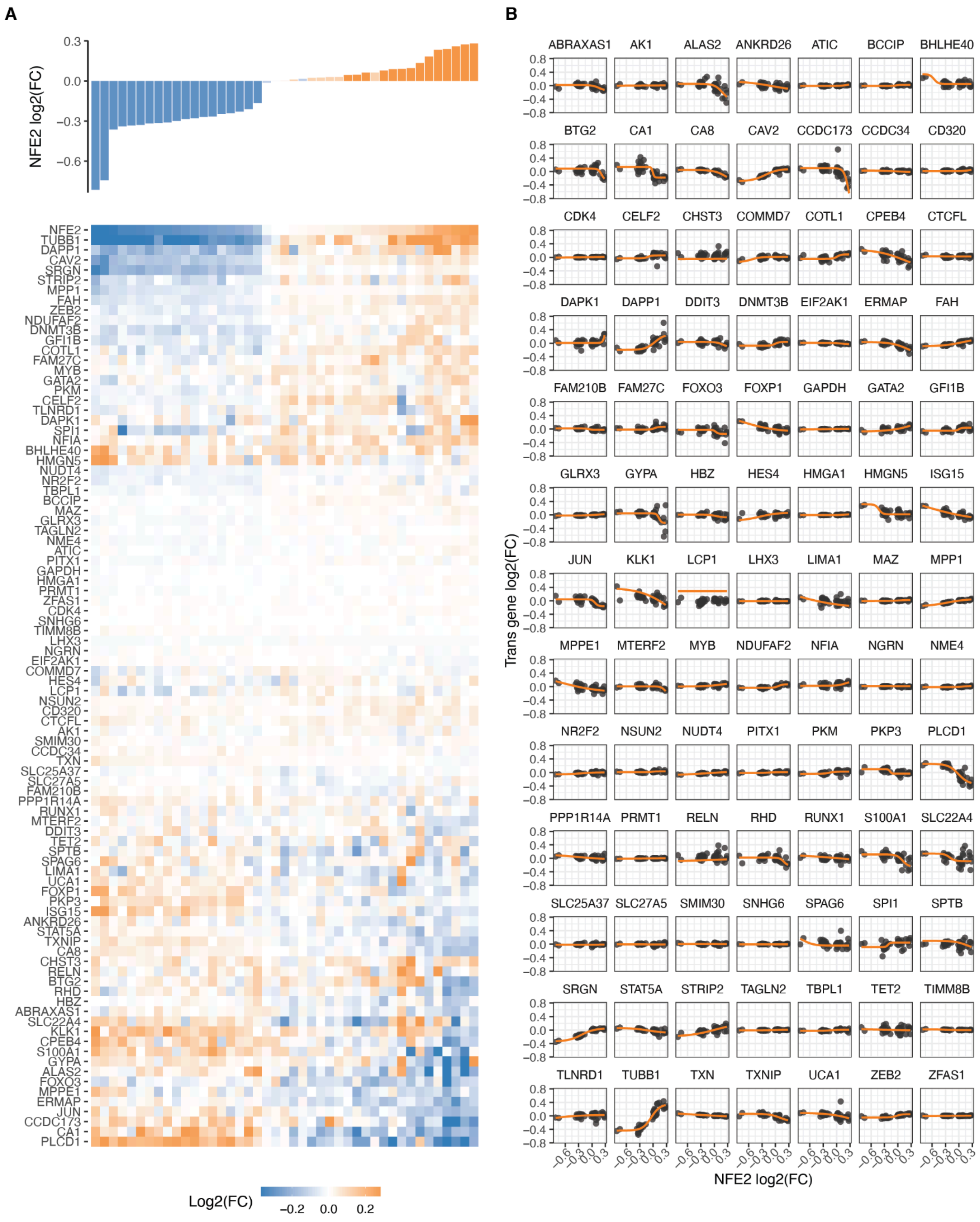

Figure S6: Transgenes responses to NFE2 dosage modulation

A. Changes in relative expression of all trans genes (bottom heatmap) in response to NFE2 expression (top barplot) upon each distinct targeted NFE2 sgRNA perturbation. The rows of the heatmap (trans genes) are hierarchically clustered based on their expression fold change linked to alterations in NFE2 dosage.
B. Dosage response curves are plotted for each trans gene against changes in NFE2 expression. The orange line represents the sigmoid model fit.

bioRxiv preprint doi: https://doi.org/10.1101/2024.03.01.582837; this version posted March 4, 2024. The copyright holder for this preprint (which was not certified by peer review) is the author/funder, who has granted bioRxiv a license to display the preprint in perpetuity. It is made available under aCC-BY 4.0 International license.

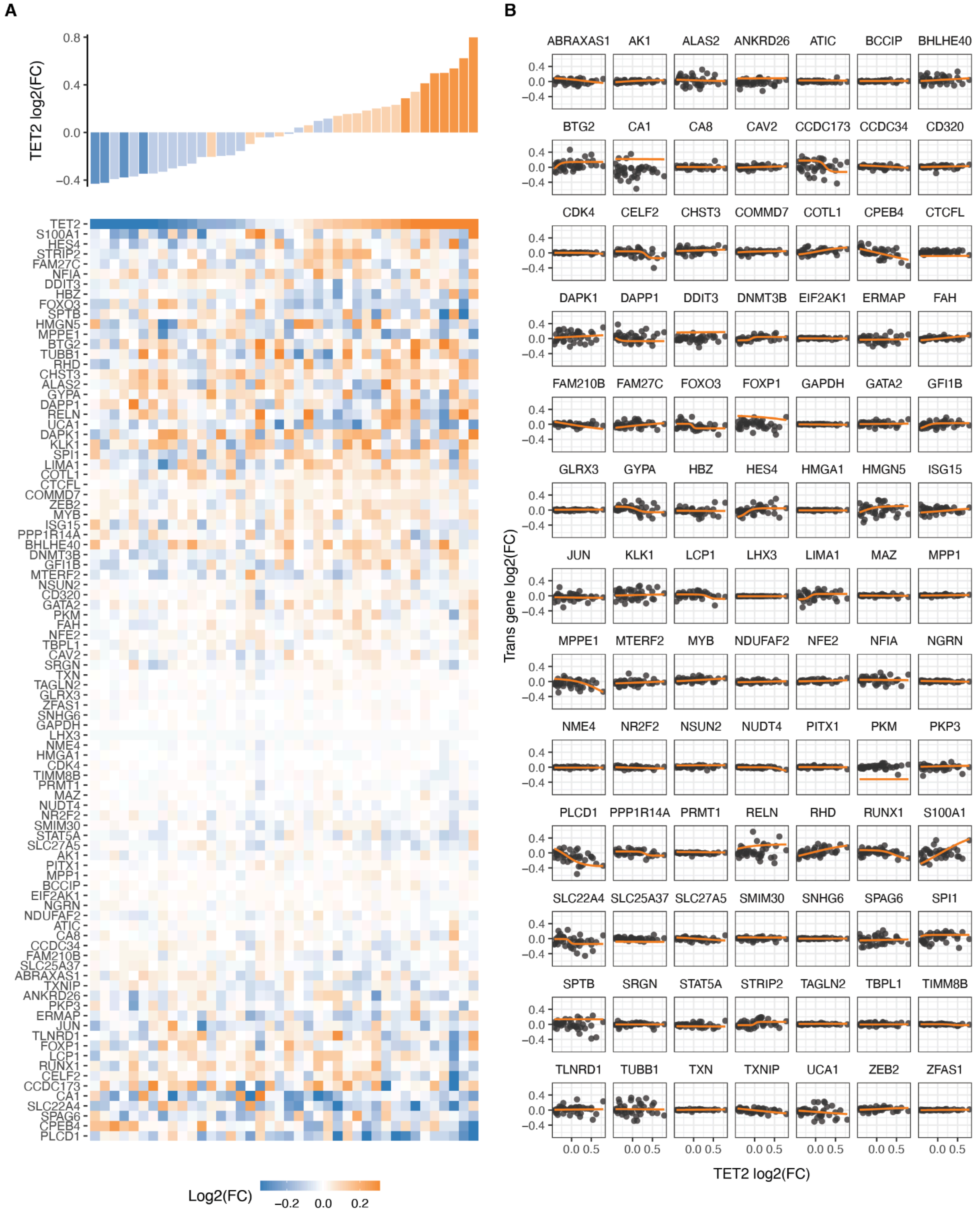

Figure S7: Transgenes responses to TET2 dosage modulation

A. Changes in relative expression of all trans genes (bottom heatmap) in response to TET2 expression (top barplot) upon each distinct targeted TET2 sgRNA perturbation. The rows of the heatmap (trans genes) are hierarchically clustered based on their expression fold change linked to alterations in TET2 dosage.
B. Dosage response curves are plotted for each trans gene against changes in TET2 expression. The orange line represents the sigmoid model fit.

bioRxiv preprint doi: https://doi.org/10.1101/2024.03.01.582837; this version posted March 4, 2024. The copyright holder for this preprint (which was not certified by peer review) is the author/funder, who has granted bioRxiv a license to display the preprint in perpetuity. It is made available under aCC-BY 4.0 International license.

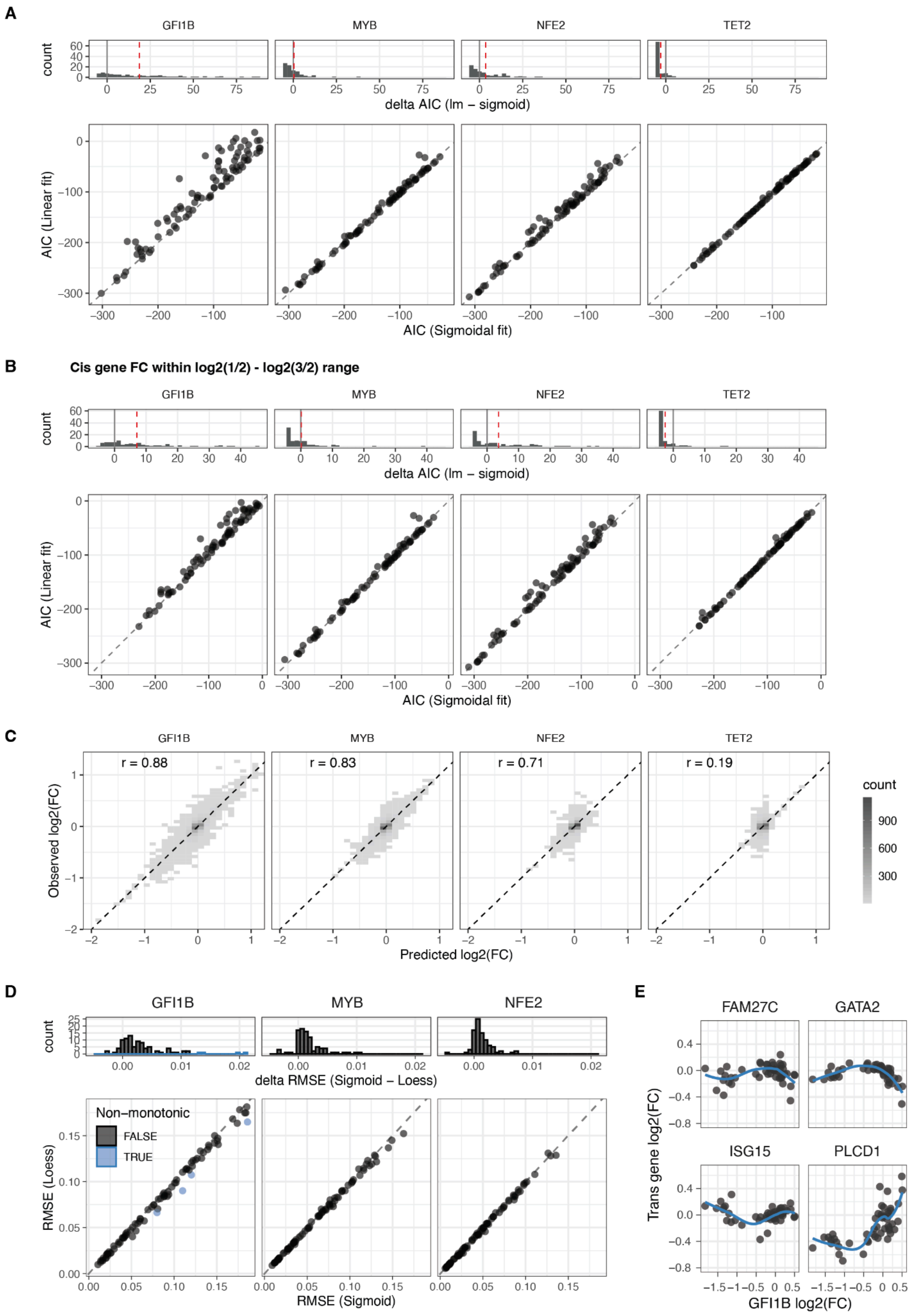

Figure S8: Dosage response linear and non-linear model fitting

bioRxiv preprint doi: https://doi.org/10.1101/2024.03.01.582837; this version posted March 4, 2024. The copyright holder for this preprint (which was not certified by peer review) is the author/funder, who has granted bioRxiv a license to display the preprint in perpetuity. It is made available under aCC-BY 4.0 International license.

A. Distribution of the difference in Akaike Information Criterion ($\Delta AIC_{linear\text{-}sigmoid}$) after fitting the sigmoidal or linear model for each trans gene based on the gradual expression perturbations of the four cis genes (top panel), and the direct comparison of the AIC of each fit (bottom panel).
B. Same as A but only fitting the models on those sgRNA perturbations that lead to a cis gene dosage change bounded between log2(1/2) and log2(3/2).
C. Agreement between observed and predicted trans genes expression fold change upon cis gene dosage modulation across a 10-fold cross-validation scheme.
D. Comparison of the Root Mean Square Error (RMSE) of the sigmoid model on the different trans genes dosage responses to the RMSE of the equivalent loess fit (bottom panel). In blue are highlighted the non-monotonic responses that correspond to the top four $\Delta RMSE_{sigmoid\text{-}loess}$ ($RMSE_{sigmoid}$ - $RMSE_{loess}$) values (top panel).

bioRxiv preprint doi: https://doi.org/10.1101/2024.03.01.582837; this version posted March 4, 2024. The copyright holder for this preprint (which was not certified by peer review) is the author/funder, who has granted bioRxiv a license to display the preprint in perpetuity. It is made available under aCC-BY 4.0 International license.

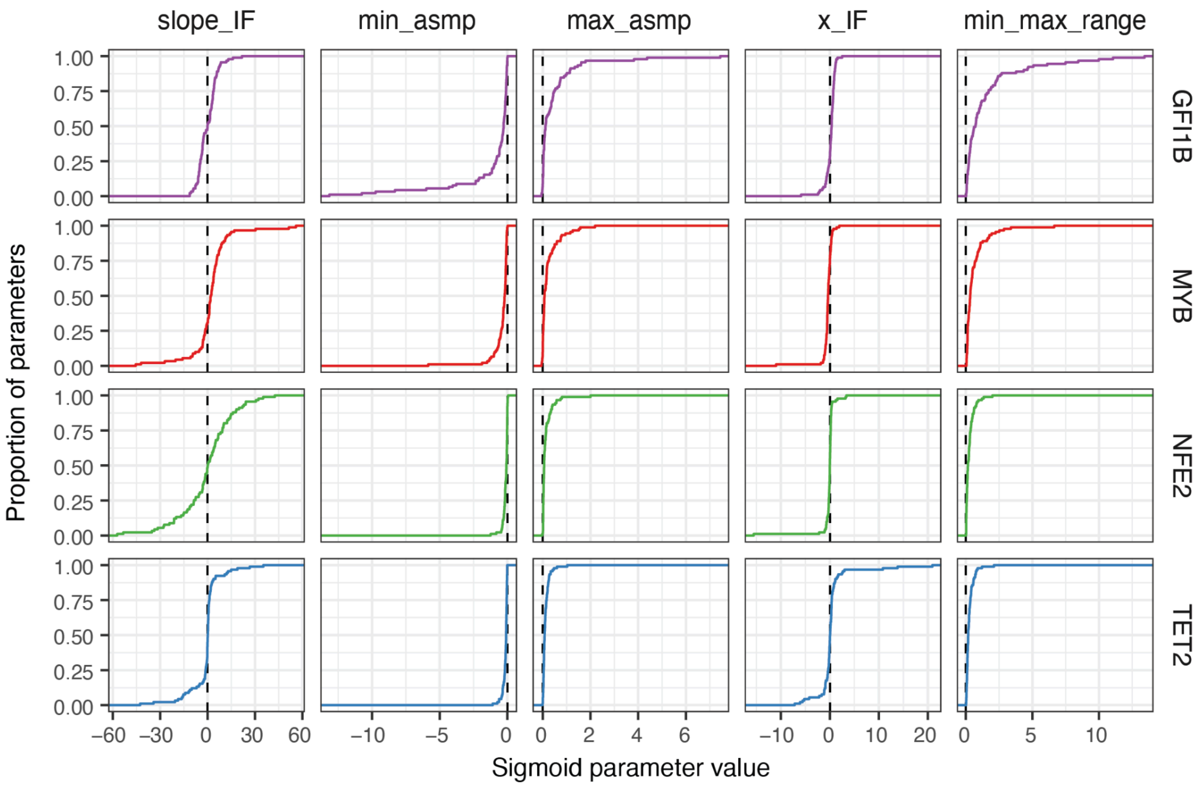

Figure S9: Distribution of the fitted parameters of sigmoidal model on dosage responses

Cumulative distribution of the four fitted parameters (first four columns) of the sigmoid model across genes given the independent perturbation of the four TFs (rows). slope_IF = slope of dosage response curve at the inflection point, min_asmp = minimum asymptote (minimum trans gene dosage level), max_asmp = maximum asymptote (maximum trans gene dosage level), x_IF = TF expression FC at the dosage response inflection point.

bioRxiv preprint doi: https://doi.org/10.1101/2024.03.01.582837; this version posted March 4, 2024. The copyright holder for this preprint (which was not certified by peer review) is the author/funder, who has granted bioRxiv a license to display the preprint in perpetuity. It is made available under aCC-BY 4.0 International license.

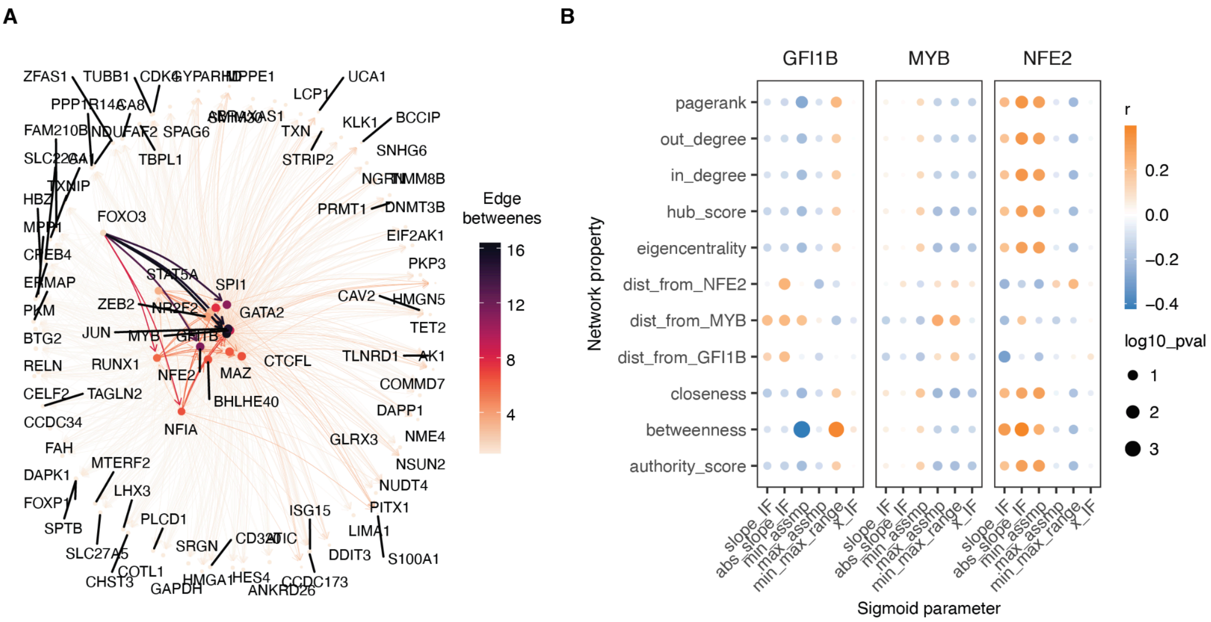

Figure S10: Relationship gene properties and TF-target network properties with TF dosage responses

A. A regulatory network constructed based on TF-target gene data Minaeva et al. 2024 with nodes and edges coloured by betweenness. Nodes are sized by their degree.
B. Heatmap illustrating the correlation between the sigmoid parameters in response to cis-gene modulation and network centrality metrics calculated based on the regulatory networks from Minaeva et al. 2024. Point size is scaled to -log10 p-value.

bioRxiv preprint doi: https://doi.org/10.1101/2024.03.01.582837; this version posted March 4, 2024. The copyright holder for this preprint (which was not certified by peer review) is the author/funder, who has granted bioRxiv a license to display the preprint in perpetuity. It is made available under aCC-BY 4.0 International license.

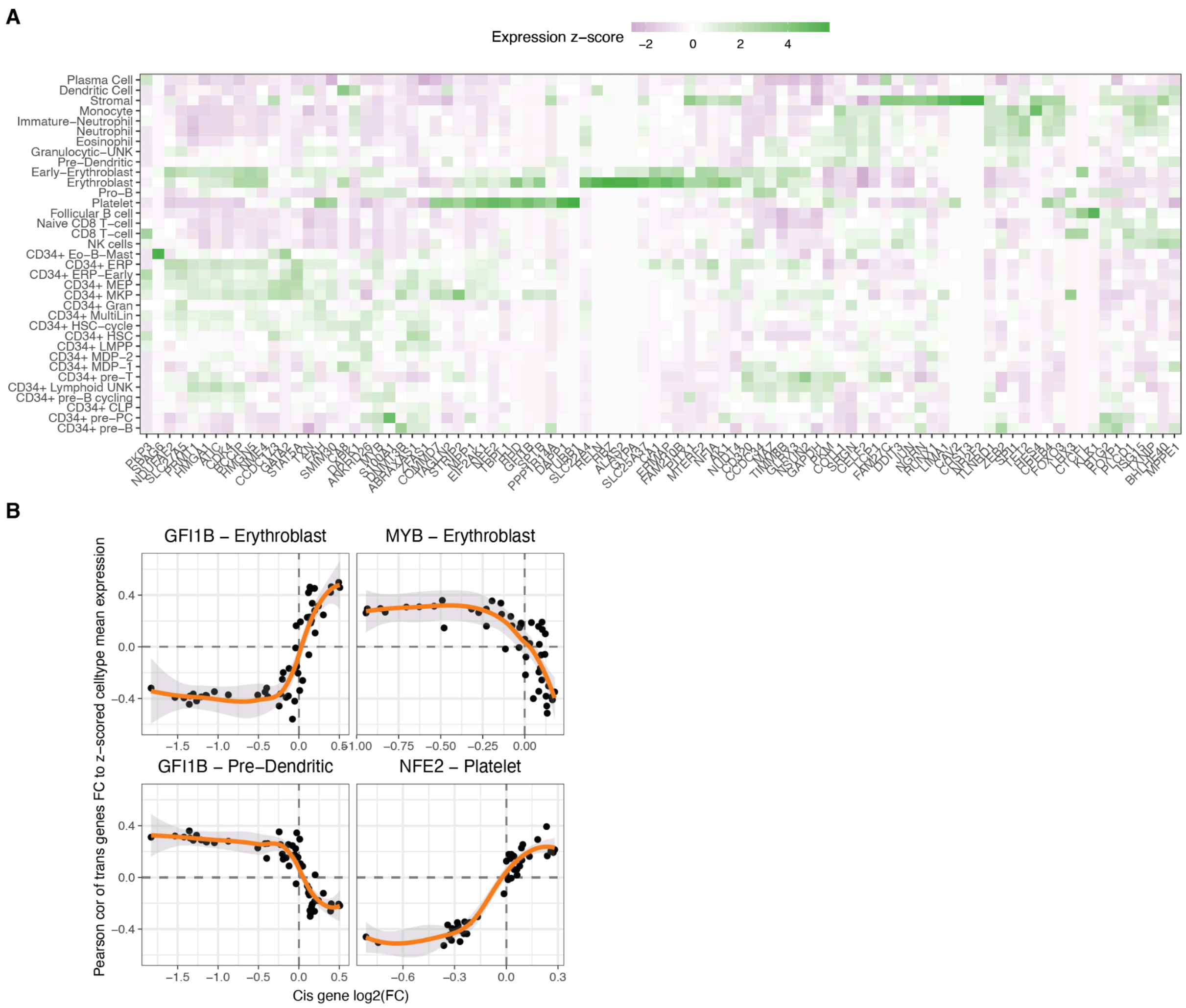

Figure S11: Transcriptional similarity among bone marrow cell types at different TF dosage levels

A. Normalised z-score mean expression across donors for targeted genes within each bone marrow cell type (Data from the Human Cell Atlas).
B. Examples of trends of correlation of transgenes expression with the TF change in dosage. The title specifies the cis gene and the cell type for which the trans effects of TF dosage modulation have been contrasted to.
